# Multidisciplinary expert panel report on fluid stewardship: perspectives and practice

**DOI:** 10.1186/s13613-023-01177-y

**Published:** 2023-09-25

**Authors:** Manu L. N. G. Malbrain, Pietro Caironi, Robert G. Hahn, Juan V. Llau, Marcia McDougall, Luís Patrão, Emily Ridley, Alan Timmins

**Affiliations:** 1 First Department Anaesthesiology and Intensive Therapy, Medical University Lublin, Lublin, Poland; 2 International Fluid Academy, Lovenjoel, Belgium; 3 Medical Data Management, Medaman, Geel, Belgium; 4 Medical Management, AZ Oudenaarde Hospital, Oudenaarde, Belgium; 5 Department of Anesthesia and Critical Care, San Luigi Gonzaga Hospital, Orbassano, Turin, Italy; 6 Department of Oncology, University of Turin, Turin, Italy; 7 Anesthesia and Intensive Care, Karolinska Institute, Stockholm, Sweden; 8 Anaesthesiology and Post-Surgical Critical Care, University Hospital Doctor Peset, Valencia, Spain; 9 Anaesthesiology, Department of Surgery, University of Valencia, Valencia, Spain; 10 Anaesthetics and Intensive Care, Victoria Hospital, Kirkcaldy, Fife Scotland; 11 Intensive Care Unit, Centro HospitalarTondela-Viseu, EPE, Viseu, Portugal; 12 UpHill Health, Lisbon, Portugal; 13 Fluid Management lead, Department of Nursing, Victoria Hospital, Kirkcaldy, Fife Scotland; 14 Pharmacy Department, Victoria Hospital, Kirkcaldy, Fife Scotland

**Keywords:** Critical care, Perioperative care, Fluid stewardship, Crystalloids, Buffered solutions, Fluid therapy

## Abstract

Although effective and appropriate fluid management is a critical aspect of quality care during hospitalization, the widespread adoption of consistent policies that ensure adequate fluid stewardship has been slow and heterogenous. Despite evidence-based guidelines on fluid management being available, clinical opinions continue to diverge on important aspects of care in this setting, and the consistency of guideline implementation is far from ideal. A multidisciplinary panel of leading practitioners and experts convened to discuss best practices for ongoing staff education, intravenous fluid therapy, new training technologies, and strategies to track the success of institutional fluid stewardship efforts. Fluid leads should be identified in every hospital to ensure consistency in fluid administration and monitoring. In this article, strategies to communicate the importance of effective fluid stewardship for the purposes of education, training, institutional support, and improvement of patient outcomes are reviewed and recommendations are summarized.

**Graphical Abstract:**

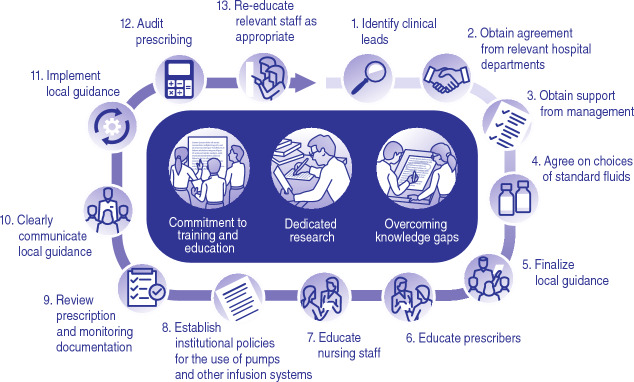

## Introduction

The primary goal of fluid stewardship is to optimize intravenous (IV) fluid administration, minimize the detrimental effects of inappropriate fluid administration, and thereby potentially improve clinical outcomes. Providing consistent educational content and rationalizing available fluids is also important. Successful institutional fluid stewardship practice is therefore a critical aspect of quality hospital care for many patients.

Despite the importance of effective fluid management as recognized by the United Kingdom’s National Institute for Health and Care Excellence (NICE) [1] and other efforts such as the British Consensus Guidelines on Intravenous Fluid Therapy for Adult Surgical Patients (GIFTASUP) [2], the Perioperative Quality Initiative (POQI) [3], and International Fluid Academy (IFA) guidance [4, 5], inappropriate fluid use continues to be an important gap in care for many institutions, with potential for impact on patient outcomes and health care costs [6–10]. Many institutions recognize that this potential care gap results in excessive costs, but this consideration is inconsistent across all organizations. Reasons cited for ongoing fluid mismanagement include poorly ingrained habits among staff and a lack of consistent training and education [4]. Targeted efforts that focus on education, recordkeeping and auditing have been shown to improve adherence to current NICE guidance [11]. Many surveys of clinicians’ knowledge have demonstrated deficits in education [11]. During the 2019 International Fluid Academy Day (IFAD) meetings held in Campinas, Brazil, and Valencia, Spain, participants (mainly physicians) were encouraged to complete a 57-question survey to investigate awareness of best practices with respect to fluid management [12]. Nearly three-quarters of participants responded that their institutions do not have any general ward or ICU guidelines for IV fluid management, and only 6.5% of respondents achieved an above-average score on knowledge testing with respect to fluid management [13].

Appropriate fluid stewardship practices are essential for optimal care in all hospitalized patients. Best practices require adequate clinician awareness, training, and education. The primary aim of this paper therefore was a fresh appraisal and discussion of the available evidence on this topic.

## Methods

A panel of leading practitioners and clinical researchers who are considered effective stewards of fluid management at their home institutions participated in a 2-day virtual Advisory Board meeting (September 15–16, 2021) to discuss the current state of fluid management practices in Western Europe and identify gaps in training and education. To ensure an ample diversity of clinical perspectives on this topic, a nurse and a pharmacist were both included on the panel. This was followed by a physical meeting held during the 10th International Fluid Academy Days meeting in Brussels, Belgium (November 26, 2021). A modified Delphi method was designed to use the collective expertise of the diverse group in answering clinically important questions and achieved consensus on several topics in accordance to the AGREE reporting checklist. This report is a result of the 3-day discussion and reflects our consensus on fluid management based on new evidence and our own individual expertise.

## Results

### Current clinical evidence and guidelines for fluid management

#### Current status on perioperative fluid management

The British GIFTASUP describe general rules about how to treat patients before, during, and after surgery [2]. The guidelines recommend that maintenance fluid consist of 1.5 to 2.5 L of water, 50 to 100 mmol of sodium, and 40 to 80 mmol of potassium daily, based upon the daily physiological needs of water and salts. The NICE organization in the UK later published a set of guidelines for fluid therapy in hospitalized adults [1]. For maintenance fluids, current NICE guidance recommends 25–30 mL of fluid and 1 mmol of sodium, potassium, and chloride per kg body weight over a 24-h period, with an adjustment using ideal body weight in obese patients. NICE guidelines for pediatric fluid administration are also available and consider children’s physiological differences in fluid and electrolyte handling [1].

These recommended amounts of fluid and sodium are rather restrictive and represent about two-thirds of what is contained in the diets of healthy Europeans [14]. However, hospitalized patients with comorbidities, and particularly the elderly, may have impaired renal function and altered handling of fluids and electrolytes, i.e., post-operatively, hence the lower recommendations in the NICE guideline. Frequent monitoring of clinical status and blood tests are essential to ensure adequate fluid provision.

While the advantages of fluid therapy are apparent in complicated surgeries, the benefits can be questioned in minor surgical procedures of short duration (< 20 min) and not associated with fluid losses [15]. Randomized clinical studies of different infusion rates suggest that uncomplicated surgery lasting 1–2 h can generally be managed with a restrictive fluid program consisting of 3–5 mL/kg/h of balanced crystalloid fluid [16]. Infusion rates lower than 2 mL/kg/h increase the risk of postoperative nausea [17]. We agree that administration of resuscitation fluid is best guided by individual flow-based hemodynamic measurements in complex patients but note that advantages are most apparent with signs of organ dysfunction in intensive care and during lengthy surgery when the hydro-electrolytic balance is unclear or complicated.

Both balanced and unbalanced crystalloid fluids are used in the perioperative period. Several large trials of more than 1000 patients have not found that saline promotes mortality or major complications (such as myocardial infarction) during surgery. Balanced crystalloid fluids are preferable as many small studies of patients and volunteers report a higher number of minor complications when 2 L or more of saline is given, such as metabolic acidosis, low urine flow, nausea, and abdominal pain [18]. The benefit of adding glucose to the IV fluid during the early postoperative phase seems to be minor at best [15].

Enhanced recovery after surgery (ERAS®) programs aim for swift recovery after surgery and recommend either near-zero fluid balance or goal-directed fluid therapy [19, 20]. Recent ERAS protocol recommendations have been published for a variety of clinical settings. In emergency laparotomy, the ERAS Society recommends immediate IV resuscitation and correction of physiologic derangements, in addition to ongoing maintenance balanced crystalloid fluid infusions [21]. In the perioperative management of patients undergoing liver surgery, balanced crystalloid solutions are recommended rather than 0.9% normal saline or colloids, citing a reduced risk of hyperchloremic acidosis and renal dysfunction, respectively [22]. The ERAS Society recommendations for postoperative management of patients undergoing gynaecological or abdominal oncology surgery include a discontinuation of IV fluids within 24 h after surgery, and a preference for balanced crystalloid solutions over 0.9% normal saline due to the risk of hyperchloremic acidosis [23]. In the perioperative management of patients undergoing total hip and total knee replacement surgery, recommendations include maintenance of fluid balance as needed to avoid over- or under-hydration [24].

Ad hoc groups have also been convened to publish consensus statements about fluid therapy. The POQI consensus statement on fundamental concepts in perioperative fluid management summarizes the goal of fluid therapy and hemodynamic management as providing the conditions that enable normal cellular metabolic function to produce optimal patient outcomes [3].

#### Current status on fluid management in the ICU setting

In the ICU setting, the NICE guidelines recommend that the need for resuscitation fluid is best determined by using individual flow-based hemodynamic measurements [1]. The Surviving Sepsis Campaign (SSC) guideline suggests that 30 mL/kg of crystalloid fluid be infused within the first 1 to 3 h in septic patients with hypotension or septic shock [23]. The benefit of early intervention is undisputed, but the fixed volume is still in question. Regarding the type of fluid, guidelines from NICE [1], GIFTASUP [2], ERAS [19, 20], and SSC [25] all support solutions other than normal saline. Aside from limited available guidelines on fluid management in the ICU setting, trials have been conducted to compare strategies and identify ideal fluid solutions. The multicenter, open-label ALBIOS trial found that albumin is a safe alternative to crystalloids, maintains kidney function, and reduces mortality in patients with septic shock [26–28]. However, we need to await the results of the ongoing ALBumin Italian Outcome Septic Shock—BALANCED (ALBIOSS-BALANCED) trial (#NCT03654001) before making final suggestions related to albumin. When deciding between a balanced solution versus saline, current evidence from trials such as BaSICS and PLUS trials reported no differences between these options in 90-day mortality [29, 30] or acute kidney injury [30], but our consensus is that a balanced solution may be preferable to 0.9% NaCl saline. A secondary post hoc analysis of the BaSICS trial using a Bayesian approach, in which new data not previously available were considered to develop new conclusions, found an association between mortality risk and the type of fluids received for resuscitation prior to study enrollment, with a benefit seen in patients who had received exclusively balanced solutions. This benefit was especially evident in patients with an unplanned hospital admission due to sepsis [31]. A secondary analysis of the SMART Clinical Trial reported a lower mortality when balanced crystalloids instead of saline solutions were administered during the hospital treatment of sepsis [32].

Guidelines on management of massive hemorrhage recommend restricting the use of crystalloids (< 3 L in the first 6 h) [33] to avoid hypervolemia [34, 35], as part of a bundle of care for patients with acute bleeding from trauma, since fluids do not have their own therapeutic benefit and should be used to “gain time”.

Guidance from the IFA stresses that perioperative fluid therapy in association with major surgery, as well as fluid therapy in intensive care, should follow the Resuscitation, Optimization, Stabilization and Evacuation (ROSE) conceptual model: liberal therapy is initially of benefit to preserve organ perfusion, and this should be followed by a more conservative or restrictive phase where fluid is withdrawn later in the process [5, 12]. More recently, the CLASSIC trial results showed that a restrictive fluid strategy is as safe as a more liberal standard fluid delivery protocol [36].

#### Knowledge gaps

Although perioperative and ICU fluid management plays a crucial role in optimizing patient outcomes before, during, and after surgery, many knowledge gaps do exist. Guidelines from organizations like NICE, IFA and ERAS provide recommendations on fluid therapy, taking into account factors such as daily physiological needs, patient characteristics, and surgery duration. While the recommended amounts of fluid and sodium may seem restrictive, they are designed to accommodate patients with impaired renal function and altered fluid and electrolyte handling. Frequent monitoring of clinical status and blood tests is essential to ensure adequate fluid provision. In the ICU setting, individual flow-based hemodynamic measurements in combination with assessment of fluid (un)responsiveness are recommended to guide the need for resuscitation fluid. Trials and consensus statements highlight the importance of choosing the appropriate fluid type, with balanced crystalloid solutions being preferable to saline due to a lower incidence of complications. Ongoing trials, such as the ALBIOSS-BALANCED trial, aim to provide further insights into the use of albumin as an alternative fluid. In cases of massive hemorrhage, the use of crystalloids is restricted to avoid hypervolemia. Overall, a personalized and phased approach to fluid therapy, transitioning from liberal to conservative strategies, appears to be beneficial in optimizing patient outcomes.

#### Group consensus

Considering current guidelines and evidence, local institutional practices, and survey findings, our advisory group consensus is that the NICE guidance provides basic knowledge but should be combined with education on clinical assessment and monitoring to guide individualized fluid management within the guideline framework, providing an understanding of fluid requirements in terms of maintenance, replacement, and resuscitation. When monitoring patients receiving fluids, daily body weight, daily and cumulative fluid balance, regular monitoring for renal function and plasma and urinary electrolyte concentrations may be used to assess volume status. Assessment of body fluid composition (e.g., with bio-electrical impedance analysis), and fluid responsiveness (e.g., with passive leg raising test) can provide fluid management guidance and other non-invasive hemodynamic monitoring tools may be of help in this setting.

In the ICU setting, the largest amount of fluids is not given for resuscitation (6%) but for maintenance (25%), nutrition (33%) and fluid creep (33%), the latter is the inadvertent (and sometimes unavoidable) administration of fluids intravenously when administering drugs or other therapies [37]. Nevertheless, most of the available literature continues to focus on resuscitation fluids. Studies now confirm the benefits of avoiding excessive 0.9% NaCl administration, including reduced risk of mortality or major complications such as myocardial infarction, in favor of balanced crystalloids, with a consensus that this is especially true in patients with sepsis, burns and diabetic keto-acidosis, but not for those with traumatic brain injury where saline should still be preferred [38]. Although the magnitude of benefit may be small in controlled studies, in the ‘real world’ volumes of fluid are not precisely controlled and many excess liters of fluid may be given. One cross-sectional, multicenter, observational study of fluid management in more than 7000 patients reported a high degree of variability in perioperative volume delivered. Moreover, advanced fluids monitoring strategies were only used in 5% and 10% of patients in the intraoperative and postoperative periods, respectively [38]. Improving basic fluid administration on medical and surgical wards is achievable with better education and standardization of use.

Reducing variability in fluid management should improve clinical outcomes particularly in terms of avoiding fluid overload/accumulation and dehydration. However, because fluid therapy is only a small part of patient’s care, studying such outcomes is fraught with difficulty, and relating outcomes such as length of stay and mortality to fluid use may be unrealistic. Fluid management in a critical care setting often involves some monitoring modalities, but even there a simple approach can go far to avoid overload and electrolyte abnormalities. The overarching purpose of fluid therapy in intensive care is to prevent organ dysfunction by maximizing organ perfusion as a tool but not a goal in and of itself.

It is important to approach all decision on fluid management and provision with the understanding that fluids are drugs, with indications and contraindications and the potential for both benefits and risks of adverse events. On this note, consistent IV fluid prescribing based on NICE or IFA guidance can be improved throughout an institution with staff education, record keeping and audits to monitor practices. Limiting the availability of specific IV fluid preparations on wards has also been implemented to improve fluid prescription practices. Patient clinical chemistry values can be tracked automatically (flagging abnormal bicarbonate, sodium, chloride, and potassium) as well as the incidence of acute kidney injury before and after the implementation of these measures [4]. Researchers found that after implementing the NICE guidelines for adults and changing the fluids used, biochemical values improved or did not deteriorate in patients before and after the change, without adverse impact on mortality or length of stay. They also stressed the time and effort required to implement these changes and noted the ongoing need for clinical judgment to guide individualized care [4].

#### Areas for further research

We propose that hospitals conduct small audit studies, focusing on patients with clear evidence of a positive cumulative fluid balance or with clinical indicators consistent with fluid overload, thereby capturing actual practice and the potential harm of fluid mismanagement. If an audit of five to ten patients demonstrates that there is a problem, much larger studies are not required before a solution is sought. At a local level, analysis of the use of fluids, can be of use in monitoring the effect of stewardship initiatives. The Belgian perspective on fluid consumption was recently presented at the 12th IFAD meeting in Brussels (https://www.fluidacademy.org/component/zoo/item/an-introduction-to-fluid-stewardship-a-belgian-perspective.html or https://fluidacademy.mn.co/posts/an-introduction-to-fluid-stewardship-a-belgian-perspective). Variations in case-mix mean that direct comparison may not be relevant, but the resulting data can highlight variations in fluids use that require action [39]. Parameters such as the amount of sodium, chloride and potassium administered with IV fluids, and the type and volume of fluid administered, and whether fluid requirements were met or exceeded can quickly identify areas for improvement. Oral fluid intake should form part of these audits as elderly patients who are not receiving IV fluids easily become dehydrated without adequate attention to oral intake. Both community-based hospitals and university/teaching hospitals should be included. The clinical complications of excess or inadequate fluid administration can be easily noted in the context of these brief audits and will demonstrate the need for change.

### Addressing the current state of fluid stewardship

#### Knowledge gaps

Knowledge gaps regarding the current state of implementation of fluid stewardship, analogous to antimicrobial stewardship, include:

- Guidelines and best practices: while guidelines exist for fluid therapy, there is a lack of standardized protocols and best practices for implementing fluid stewardship programs.
- Metrics and monitoring: there is a need for standardized metrics and monitoring tools to assess the impact of fluid stewardship interventions. Currently, there is a lack of consensus on the most effective metrics to evaluate fluid management practices and outcomes.
- Education and awareness: the level of education and awareness among healthcare professionals regarding fluid stewardship is not well-established. Similarly, homogeneity on the role of fluid education within the track of school of medicine programs is not well-established.
- Implementation strategies: limited research exists on implementation strategies for fluid stewardship programs. There is a need to identify effective strategies for integrating fluid stewardship into clinical workflows, fostering interdisciplinary collaboration, and overcoming barriers to implementation.
- Data collection and analysis: the collection and analysis of data related to fluid management practices are currently inconsistent.
- Impact on patient outcomes: the impact of fluid stewardship on patient outcomes, such as morbidity, mortality, and hospital-acquired complications like electrolyte and acid–base disturbances, or fluid accumulation syndrome, is not well-established.
- Economic considerations: the economic implications of fluid stewardship, including cost-effectiveness and resource utilization, have not been extensively studied.
- Multi-center studies: there is a paucity of multi-center studies examining the implementation and outcomes of fluid stewardship programs across different healthcare settings.

Addressing these knowledge gaps will provide valuable insights into the current state of fluid stewardship implementation and guide the development of evidence-based strategies to optimize fluid management practices, improve patient outcomes, and ensure the responsible use of fluids in healthcare settings.

#### Group consensus

While effective fluid stewardship should be a cornerstone of good medical practice in hospitalized patients, insufficient efforts have been made to track patient outcomes with good fluid stewardship, and conversely the clinical implications of poor fluid stewardship. Evidence is especially lacking in the setting of pre-operative care, and the care of patients in general hospital wards. The complications associated with poor fluid management can include organ hypoperfusion or overload, renal dysfunction, overall fluid overload/accumulation and disturbances of acid–base or electrolyte balance. While inconsistently documented in the current literature, these complications are likely to impact hospital or ICU length of stay, long-term organ function, all-cause mortality, and quality of life. For these reasons, we recommend an approach to institutional fluid stewardship that includes mechanisms for tracking indicators of good compliance and clinically relevant patient outcomes in those who receive in-hospital fluids.

When tracking fluid usage for the purpose of assessing appropriate use and patient outcomes, institutions should consider the following metrics:

- Quantity of fluids delivered (total amount, per indication, expressed as liters per hospital admission and liters per occupied bed day)
- Type of fluids delivered (colloids versus crystalloids, balanced versus unbalanced, hypotonic versus isotonic, fluid composition, etc.)
- Timing of administration
- Rate of administration
- Duration of administration
- Route of administration
- Potential factors contributing to fluid dilution (drugs, fluid creep, etc.)
- Presence and potential role of total salt and fluid loading or accumulation

#### Areas for further research

Based on our collective experience in institutions in Western Europe, we agree that there are multiple areas for practice improvements, and that some improvements may be guided by data-driven answers to lingering clinical questions. For instance, in the perioperative setting, there are evidence gaps on whether restrictive or liberal fluid administration policies are more effective, and how closely fluid resuscitation should be tied to mean arterial pressure or organ perfusion pressure. The ideal type of fluid in this setting is also an area of continued debate. In emergency and ICU settings, evidence is still lacking to guide consistent care with respect to fluid type, restrictive versus liberal fluid delivery, and the ideal management of high-risk patients. In the general hospital setting, the most important questions center on best practices for implementing and maintaining effective fluid stewardship programs. Further research is needed to develop comprehensive guidelines that encompass all aspects of fluid management, including clinical assessment of fluid status and fluid (un)responsiveness, body composition and hemodynamic monitoring, and appropriate use. The focus should be on identifying adequate indicators and monitoring strategies for evaluating the success of fluid stewardship programs. Research is required to determine the most effective educational interventions to improve knowledge and understanding of optimal fluid management practices. This includes developing training programs for healthcare professionals at all levels of care and to establish standardized methods for data collection and analysis, including the use of electronic health records, to facilitate benchmarking, quality improvement, and research in fluid stewardship. Finally, in all settings, improved tracking of data on the efficacy of fluid management practices, fluid-associated complications, and the cost-effectiveness of fluid stewardship are needed to confirm and implement best practices.

### Costs associated with current practice

#### Knowledge gaps

There are several knowledge gaps regarding the costs implicated in current practice on fluid management. Firstly, there is a lack of comprehensive cost-effectiveness studies that evaluate different fluid therapy interventions, hindering our understanding of the economic value of these interventions. Additionally, limited research has been conducted on the actual resource utilization and wastage (e.g., plastic bags) associated with fluid management practices, preventing a clear assessment of the financial and environmental implications. The economic burden associated with fluid-related complications, such as fluid accumulation, electrolyte imbalances, and organ dysfunction, has not been extensively studied, making it difficult to quantify the costs involved in managing these complications. Furthermore, there is a need to evaluate the cost implications of adhering to fluid management guidelines, which can provide insights into the potential cost savings and resource optimization. Limited research also exists on the economic impact of implementing fluid stewardship programs in healthcare settings, highlighting the need for studies that assess the costs associated with program implementation and potential return on investment. Lastly, there is a lack of comprehensive health economic models specific to fluid management, which could provide a more holistic understanding of the financial aspects involved. It is important to recognize that while fluid therapy may be perceived as financially insignificant, the reality is that it requires significant resources. Factors such as the cost of fluids, administration sets, infusion pumps, but also training, auditing, and monitoring contribute to the overall financial burden. Raising awareness of these costs may serve as a financial incentive to improve fluid management practices beyond clinical justifications.

#### Group consensus

Wide variations in prices for fluids and consumables in different countries make overall fluid administration cost comparisons challenging. In addition, economic circumstances may influence the degree to which staff, regular audits and pumps can be used to influence good stewardship. However, our collective experience often suggests that substantial financial savings are possible with the effective use of fluids through implementation of consistent fluid stewardship [4]. These savings, coupled with the morbidity risks demonstrated with poor use of fluids [1], suggest that the prevention of poor fluid use is likely to reduce costs. Further research is warranted to determine the comparative costs of effective versus poor fluid management in various hospital settings.

In summary, outcomes and their associated cost need to be assessed across the full hospital stay, not just in individual wards, and should include data on the use of fluids linked with data relating to adverse outcomes such as increased length of stay and need for further intervention. In addition, consistent definitions are needed for complications and the severity of potential adverse outcomes related to fluid management practices. In many cases, necessary data are already being collected for different purposes. Therefore, new methods of managing such data need to be developed to demonstrate the benefits of appropriate fluid stewardship.

#### Areas for further research: big data

Poor fluid management may not directly result in mortality but can impact clinically relevant outcomes such as acid–base and electrolyte disturbances, fluid accumulation and acute kidney injury, that in turn may contribute to morbidity and mortality. Considering the risk for poor outcomes, it is important for institutions to capture the potential cost benefit of good fluid stewardship. To achieve this analysis, institutions will need to rely on big data strategies derived from available data sources as opposed to snapshots of local cost data. These data sources already exist and include prescription data, laboratory records, and patient administration and information systems. In the meantime, without robust data on complication rates, a real cost-effectiveness analysis might be extremely challenging, and the best approach that can be applied is to monitor fluid use and outcomes in specific hospitals. One such example of real-world data is the European Health Data & Evidence Network (EHDEN) project (https://www.ehden.eu/), which aims to collect Observational Medical Outcomes Partnership-Common Data Model compatible (OMOP-CDM) data from 100 million patient records [40]. Developing models that consider costs, outcomes, and long-term implications of different fluid management approaches can assist in decision-making, resource allocation, and could result in potential cost savings associated with implementing evidence-based fluid management practices.

### Strategies for improving training, education, and institutional fluid stewardship

#### Training and education

Significant gaps currently exist in consistent education and training on fluid management, and innovative solutions are needed to drive change and transformation. Institutions should therefore implement educational programs on fluid stewardship to achieve their quality improvement and patient safety goals. The fundamental concept of this education should be that the goal of IV fluid therapy is to “restore and maintain tissue fluid and electrolyte homeostasis, and central euvolemia” [41] with an understanding of proper fluid management goals in resuscitation, replacement, nutrition and maintenance settings. Comprehensive educational programs (CEP) should be designed with the goals of (1) improving and expanding training; (2) involving clinicians of multiple specialties, including nurses and pharmacists; (3) using evidence-based medicine and guidelines; (4) providing adequate resources to widely disseminate key practice recommendations.

The NICE fluid guidelines provide a starting point for an educational program [1]. Educational components should include:

1. Understanding the physiology of water and electrolytes homeostasis.
2. Knowing the risk, benefit, and harm of IV fluids.
3. Assessing fluid and electrolyte needs.
4. Assessing fluid and volume status and fluid (un)responsiveness.
5. Prescribing IV fluids properly to each patient.
6. Evaluating and documenting changes.
7. Monitoring the response to IV fluids.
8. Taking further action as required.
9. Reporting complications of fluid management or administration as incidents that require investigation, to provide a basis for learning and improvement.

When planning education, it is essential to provide a platform that meets the needs of all healthcare practitioners at all stages of their careers. In one questionnaire-based analysis of clinicians responsible for fluid management, with experience levels ranging from trainees to experienced clinicians, fluid management knowledge scores were low and most participants reported having experienced unreported fluid-related serious adverse events [42].

To reach all clinicians involved in fluid management with education and training based on evidence-based medicine and guidelines, educators should abandon old ideas that are often based in silo thinking and understand the value of a system approach to education that improves patient care. A systems approach recognizes the roles that all specialties play in patient care and encourages open communication that avoids practices that unnecessarily separate different aspects of the fluid management team into independent component parts. To achieve this systems approach goal and to maximize staff involvement from all relevant specialties involved in fluid management, effective training should take advantage of a variety of innovative educational resources as summarized in Table 1.

**Table 1:** Potential resources for fluid management education

Training tools	Educational venues	In-hospital education
• Clinical simulators ∘ Interactive algorithms embedded in decision support systems • e-Learning platforms ∘ Smartphone-based applications • Innovative technical training platforms ∘ Gamification of fluid training	• Educational programs endorsed by national and/or international congresses ∘ Live and virtual symposia • Virtual or live webinars ∘ Train-the-trainer events Peer-to-peer teaching • Dedicated fluid conferences International Fluid Academy Day (https://www.fluidacademy.org/)	• Institutional fluids ambassador identification and specialized training ∘ Train-the-trainer events • Internal multidisciplinary fluids training events ∘ Peer-to-peer teaching ∘ Specialized departmental presentations • Posters and other learning tools ∘ Ward-specific educational boards ∘ Pocket guides

Better education and training require a transformation in mindset and behavior among both junior and established clinicians. It is important to identify fluid stewards and institutional ambassadors who support not only staff education, but also change and who can obtain necessary buy-in from hospital administrators, such as medical directors. Innovative virtual learning platforms should be considered for staff convenience and flexibility. Targeted education should be provided to all staff responsible for fluid management decisions.

Guidelines are valuable tools to ensure consistent fluid delivery practices after regular staff education is established. Any proposed guideline should include assessment, prescription, monitoring, fluid balance charting and regular review of clinical status.

There are several opportunities for future research to optimize training and education in fluid stewardship. First, studies are needed to evaluate the effectiveness of different training and education methods and materials such as online courses, in-person training, gamification, virtual and augmented reality technologies and hands-on simulations, to enhance the learning experience and improve retention of information. Second, such studies should assess the effectiveness of training and education programs in promoting long-term behavior change and improved fluid stewardship practices. Third, studies should strive to identify the best practices for implementing training and education programs in different organizational settings and the potential benefits of collaboration and information sharing between organizations for improving fluid stewardship practices. Finally, it is important to better understand the impact of leadership and management on the effectiveness of training and education programs.

**Fig. 1:**
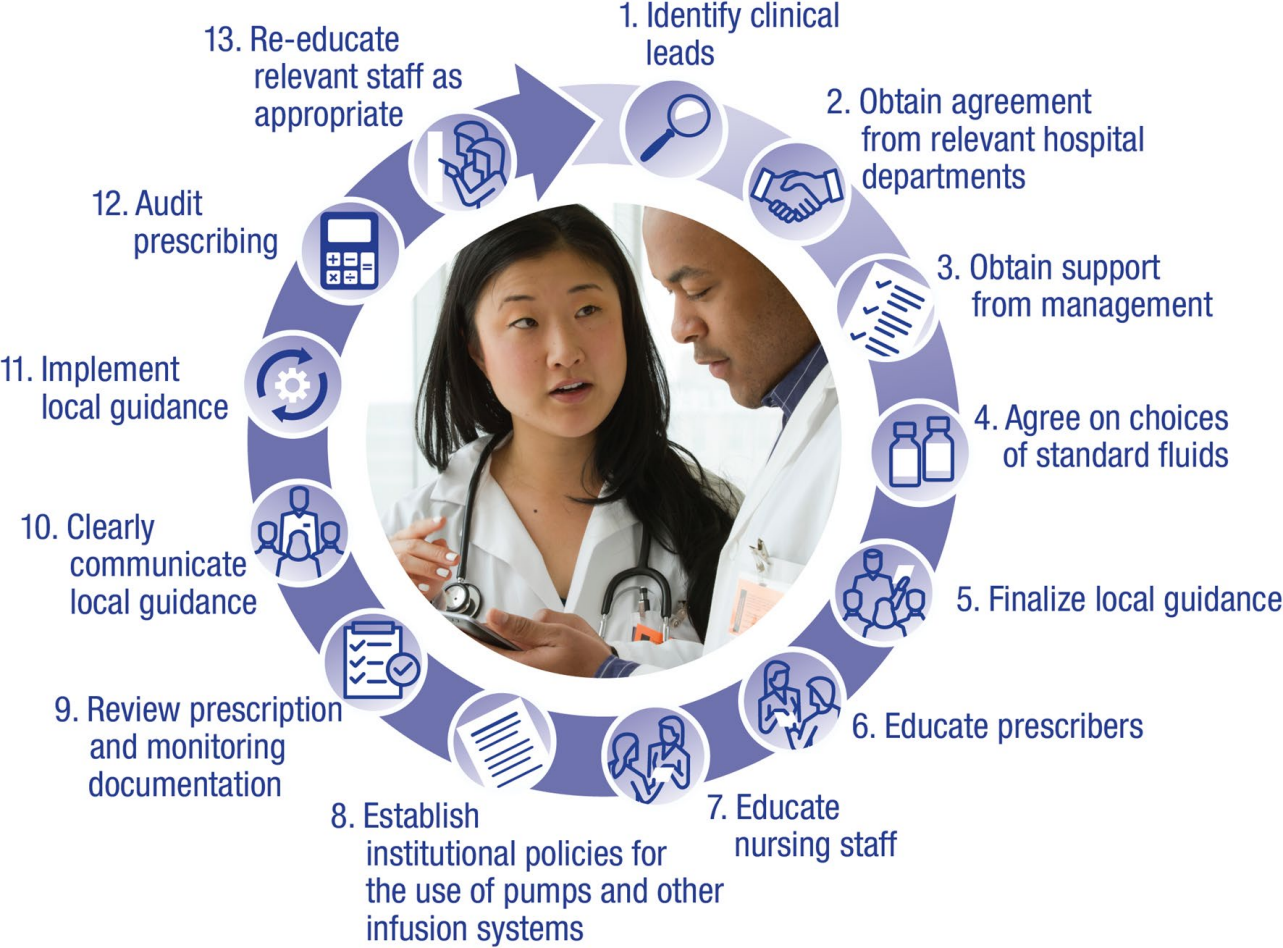
Strategies to achieve institutional best practices in fluid stewardship

Current fluid management training and education remains insufficient, and government advocacy may be needed to increase attention to best practice in this area. Adequate staff training requires planning and can be achieved through dedicated learning days or other strategies, but it needs to be ongoing. To ensure that any educational program is truly comprehensive, new technologies and multiple platforms should be employed, both to maximize impact and to provide avenues for attendees to connect and share ideas on best practices. For review, a summary of this panel’s recommendations is available in Table 2 and Fig. 1.

**Table 2:** Summary of expert consensus on intravenous fluid management

Group consensus	
Challenges	Suggested solutions
Best practices are available but regular staff training and institutional buy-in is necessary to consistently achieve better fluid management practices	Develop institutional strategies and protocols, including education, to improve adherence to current guidelines Limit availability and usage of specific IV fluid preparations (multi-electrolyte solutions) based on clinical setting to reduce confusion and errors Use key performance indicators to track fluid management practices
Consistent fluid management education may be locally inconsistent	Position fluid management as an interesting topic, and as a pharmacologic prescription (similar to antibiotic stewardship practices) Address distinct fluid delivery strategies for resuscitation, replacement, and maintenance therapy Expand patient and/or caregiver education in order to emphasize the importance of appropriate fluids and to track symptoms Standardize the approach to fluid prescription and fluid use across a country or countries Select fluid ambassadors to lead and disseminate the knowledge Consider posters, print sheets, e-learning opportunities, live learning events, podcasts, or symposia Consider new technology such as patient simulators for fluid management
Institutional buy-in and oversight is needed to implement and maintain effective fluid stewardship	Identify fluid stewards and ambassadors with effective leadership and communication skills Track outcomes to demonstrate the cost-effectiveness of appropriate fluid stewardship

*IV* intravenous

#### Institutional fluid stewardship

The practice scope of health care providers involved in fluid management varies widely, from the complicated and risky administration and monitoring of fluids in the critical care setting to procurement, quality improvement and evidence-based research projects.

Monitoring (daily and cumulative) fluid balance is an integral component of fluid therapy and good patient care; it can identify potential problems and allow for earlier escalation when required. A trigger point on a fluid balance chart that supports fluid delivery decision-making is important for the identification of suboptimal or increased fluid intake or output. These trigger points should be highlighted in an institution-specific educational program that emphasizes the importance of early warning scores (EWS) and strategies for an appropriate response. Patient information leaflets encourage patients and their relatives to be aware of their fluid needs and explain IV therapy. Self-monitoring of intake is possible for some patients.

**Fig. 2:**
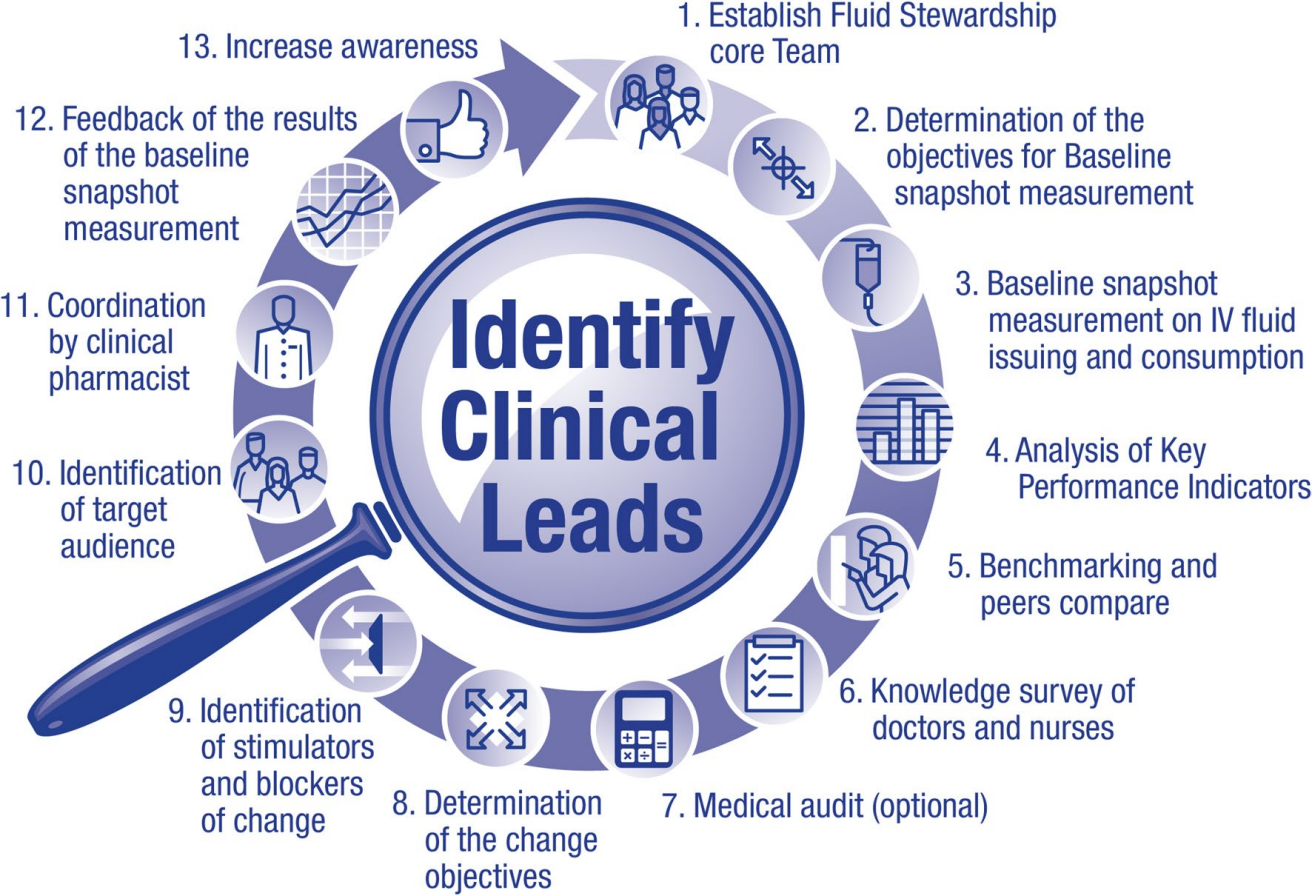
Strategies to engage clinical leads with implementation of a fluid stewardship program

A consistent approach to teaching fluid therapy based on established guidelines, as well as strong fluid stewardship, should help reduce prescriber confusion when faced with the need to prescribe fluids in different patient scenarios. As we have discussed, all institutions should consider a commitment to effective fluid stewardship at the local level. Institutions that have yet to implement standardized fluid stewardship should consider the following steps for success (Fig. 1):

1. Identify clinical leads within the organization, including medical (usually overall lead), nursing, and pharmacy staff (Fig. 2).
2. Obtain agreement from relevant clinical areas of the organization, e.g., anaesthetics and ICU; renal medicine; endocrine medicine; gastro-intestinal medicine and nutrition; other medical specialties; general surgery; orthopedics; and maternity.
3. Obtain agreement and support from organizational management (medical director, medical council).
4. Agree on choices of standard fluids. Ensure suitable arrangements in pharmacy, and appropriate stock levels on wards.
5. Finalize local guidance with the core fluid team.
6. Educate prescribers, including those who are in training.
7. Educate nursing staff.
8. Establish institutional policies for the use of pumps and other infusion systems, and ensure that adequate equipment is available.
9. Review prescription and monitoring documentation.
10. Clearly communicate local guidance.
11. Implement local guidance.
12. Audit prescribing, administration and clinical issues related to fluids, and provide feedback to relevant staff on all wards.
13. Re-educate relevant staff as appropriate, and consider amending policies in response to observed challenges.

Once fluid stewardship is implemented, metrics for recording education for relevant staff should include number of learners taught and accessing e-learning modules, assessment results, and ultimately whether prescribers are following guidelines, as determined by information from snapshot audits and fluid usage data (Fig. 2).

#### Best practices for fluid stewardship

For the attending clinician, the process of fluid prescription may be condensed to 4 questions:

1. Does my patient need fluid or is there a benefit of fluid administration? Remember that the best fluid may be the one that has not been administered unnecessarily.
2. If so, why? i.e. Is it for maintenance, replacement of losses, or resuscitation, or do they require fluid restriction? Is there body compartment redistribution?
3. Which fluid should be used in these differing scenarios?
4. How much should I give to the patient, when, and for how long (dosing, rate, speed, timing, duration, route of administration)?

After starting an IV fluid, the next questions that should be addressed are:

1. When to stop IV fluids? When shock has been resolved. This question addresses the risks of ongoing fluid administration.
2. When to start fluid de-escalation? (e.g., when to stop maintenance fluids or when to start hypercaloric enteral feeding to reduce fluid intake and the risk of fluid accumulation)?
3. When to start fluid removal (deresuscitation)? When the presence of fluid accumulation or global increased permeability syndrome has a negative impact on end-organ function. This question addresses the benefits of fluid removal (e.g., improvement of pulmonary edema).
4. When to stop fluid removal? This question addresses the risks of fluid removal (e.g., hypoperfusion)

To expand on these questions, consider the “Five P’s” of effective fluid prescriptions:

- Prescriber: makes a clinical decision regarding fluid management.
- Prescription: is written, accounting for Drug, Dose and Duration.
- Pharmacist: checks the prescription for inconsistencies.
- Preparation: the prescribed fluid is prepared with any necessary additions (e.g., electrolytes).
- Patient: the fluid is administered to the patient; the process, response and follow-up management is handled by fluid stewards.

Finally, all staff responsible for fluid management should regularly monitor patients for the appropriateness of fluid prescriptions, including initial patient assessment, decisions on fluid indication, fluid prescription, and regular fluid management. These stages for checking on the appropriateness of IV fluid therapy are summarized in Table 3 [43].

**Table 3:** Four stages of monitoring the appropriateness of fluid prescription at the bedside. Adapted with permission from [43]

Stage of evaluation	Audit standard
1. Assessment	• The patient’s fluid balance is assessed on admission in the hospital • Daily as well as the cumulative fluid balance is calculated • The patient’s fluid and electrolyte needs are assessed as part of every ward review • The assessment includes the use of an appropriate clinical parameter for evaluation of the fluid balance • Patient’s body weight is measured • Body composition and volume excess are accessed with bio-electrical impedance analysis • Signs and symptoms for fluid accumulation are daily screened • Hemodynamic monitoring is performed • Recent laboratory result with urea and electrolytes (within 24 h of fluid prescription) • Urine analysis
2. Indication	A. Resuscitation • For patients in need of fluid resuscitation: ∘ The cause of the fluid deficit is identified ∘ An assessment of shock or hypoperfusion is made ∘ A fluid bolus of 4 mL/kg of crystalloids is given • Patients who have received initial fluid resuscitation are reassessed ∘ Dynamic assessment of volemia parameters before AND after fluid bolus (e.g., CVP, fluid responsiveness, PPV, SVV, passive leg raising test) • Care is upgraded in patients who have already been given > 2000 ml or 30 ml/kg (whichever comes first) of crystalloids and still need fluid resuscitation after reassessment • Patients who have not had > 30 mL/kg of crystalloids and who still need fluid resuscitation after reassessment receive 250–500 mL of crystalloids and have a further reassessment
	B. Maintenance • If patients need IV fluids for routine maintenance alone, the initial prescription is restricted to: ∘ 25–30 mL/kg/day (1 mL/kg/hr) of water and ∘ Approximately 1 mmol/kg/day of potassium (K^+^) and ∘ Approximately 1–1.5 mmol/kg/day of sodium (Na^+^) and ∘ Approximately 1 mmol/kg/day of chloride and ∘ Approximately 50–100 g/day (1–1.5 g/kg/day) of glucose to limit starvation ketosis • Definition of inappropriateness in case of electrolyte disturbances ∘ Solutions not containing adequate amount of sodium in case of hyponatremia (Na < 135 mmol/L) ∘ Solutions not containing adequate amount of potassium in case of hypokalemia (K < 3.5 mmol/L) ∘ Solutions containing too much sodium in case of hypernatremia (Na > 145 mmol/L) ∘ Solutions containing too much potassium in case of hyperkalemia (K > 5 mmol/L)
	C. Replacement, redistribution and creep • If patients have ongoing abnormal losses or a complex redistribution problem, the fluid therapy is adjusted for all other sources of fluid and electrolyte losses (e.g., normal saline may be indicated in patients with metabolic alkalosis due to gastro-intestinal losses) • All sources of fluids administered need to be detailed: crystalloids, colloids, blood products, enteral and parenteral nutritional products, intravenous medication and oral intake (water, tea, soup, etc.) • Precise data on the concentrated electrolytes added to these fluids or administered separately need to be documented • Fluid creep is defined as the sum of the volumes of these electrolytes, the small volumes to keep venous lines open (saline or glucose 5%) and the total volume used as a vehicle for medication
3. Prescription	• The following information is included in the IV fluid prescription: ∘ The type of fluid ∘ The rate of fluid infusion ∘ The volume of fluid • The estimated duration of fluid administration • The IV fluid prescription is adapted to current electrolyte disorders
4. Management	• Patients have an IV fluid management plan, including a fluid and electrolyte prescription over the next 24 h • The prescription for a maintenance IV fluid only changes after a clinical exam, a change in dietary intake or evaluation of laboratory results

*CVP* central venous pressure, *IV* intravenous, *PPV* pulse pressure variation, *SVV* stroke volume variation

In addition to our recommendations and those we have reviewed, there are multiple resources available to support staff who are responsible for instituting and maintaining effective fluid stewardship practices within their organizations:

- International Fluid Academy (IFA): https://www.fluidacademy.org/ and also via the new portal https://fluidacademy.mn.co. The IFA offers program details, presentations, and other resources from past and upcoming IFA Day (IFAD) events. IFA also maintains a blog through the site on relevant clinical and practice topics [44, 45].
- Turning the Tide: https://criticalcarenorthampton.com/turning-the-tide/. Offered by Critical Care Northampton, this resource offers “useful downloads and resources in order to promote excellence in the field of IV fluid safety. The section is run and managed by various UK experts within the field, hailing from backgrounds within Intensive Care, Anaesthetics, Specialist Nursing and pharmacy, to name a few.” [46].
- Fluid Stewardship (East Lancashire Hospitals). https://elht.nhs.uk/services/pharmacy/fluid-stewardship. This online resource offers brief videos that review the concept of fluid stewardship, fluid resuscitation, routine maintenance fluids, fluid replacement and redistribution, and fluid balance [47].

### New technology and digital tools for training in fluid stewardship

Multiple researchers and clinicians have embedded physiologic models for fluid management in complex simulation software. The SAPHIR (Systems Approach for PHysiological Integration of Renal, cardiac and respiratory function) project approached the integration of renal, cardiac, and respiratory function systems, with circulating volume playing an important role [48]. The work of the SAPHIR was an early example of integrating physiologic models with complex simulation software and is not necessarily used in fluid management training. Subsequent to this work, Dingley et al. assessed the usability of an electronic device for fluid dosage calculation in pediatric burn patients, finding that this process was safer and faster than non-electronic methods for fluid calculations [49]. Bodger et al. came to similar conclusions, although the need to cross-check with nomograms was emphasized [50]. However, tools that help identify the right fluid in the right setting for the right patient are still scarce in the clinical setting. Among the tools that are currently available are interactive algorithms embedded in decision support systems. Care automatization through such decision support systems, assuming full interoperability with electronic patient records, will be valuable for effective fluid stewardship when widely adopted. In terms of high-fidelity simulation, a widely used tool for medical training, the technology of current physiologic models does not allow for different interactivity for every fluid delivered, namely for resuscitation simulations. This is an opportunity for improvement in this rapidly growing field of clinical training support.

High-fidelity simulations and other opportunities for in situ experiences could be valuable tools for fluid management training. As innovation continues and younger populations enter medical training, new options like gaming-based platforms may also be explored to further promote the potential for technology-based education.

Gamification or serious games for training has been used in other clinical areas with success, and appears to be at least as effective as traditional learning, and may be more effective for improving knowledge, skills, and trainee satisfaction [51]. However, research on the role that these tools might play in fluid training is still lacking.

## Conclusions

Fluid stewardship is defined as a series of coordinated interventions, introduced to select the optimal fluid, dose, rate and duration of therapy that results in the best clinical outcome, prevention of adverse events, and cost-effectiveness. Guidelines are available that standardize fluid management, and effective fluid stewards can be identified for every hospital ward to ensure consistency in delivery and monitoring. Fluid use data and biochemical markers may be used to maintain hospital administration support for training, education, and standardized fluid management protocols. Clinical outcomes data may be used on a small scale to demonstrate the value of fluid prescription. Finally, innovative e-learning and electronic simulation tools are increasingly available to support training and education. Rather than awaiting further research results, it is advisable to take proactive action and immediately initiate and implement fluid stewardship in your hospital and ICU.

## Take-home message

Recent surveys and evidence of practice gaps suggest additional education is needed on the appropriate administration, timing, dosage, duration, rate, de-escalation, and monitoring of fluids in all hospital settings and wards. Therefore, effective fluid stewards should be identified in every hospital to ensure consistency in fluid administration and monitoring. We describe best practices for fluid stewardship and review strategies to communicate the importance of effective fluid stewardship programs for the purposes of education, training, institutional support, and improvement of patient outcomes.

## Data Availability

Data sharing is not applicable to this article as no datasets were generated or analyzed during the current study.
